# Factors influencing medical students’ interest in obstetrics and gynecology: a longitudinal study on career preferences

**DOI:** 10.1007/s00404-024-07875-7

**Published:** 2025-01-07

**Authors:** Lina Duhm, Agnes Wittek, Adeline Walter, Ruben Plöger, Nicolas Haverkamp, Milka Marinova, Brigitte Strizek, Florian Recker

**Affiliations:** 1 Present Address: Department of Obstetrics and Prenatal Medicine, University Hospital Bonn, Venusberg Campus 1, 53127 Bonn, Germany; 2 Office of the Dean of Studies, Faculty of Medicine Bonn, Rhenish-Friedrich-Wilhelms-University Bonn, Venusberg Campus 1, 53127 Bonn, Germany; 3 Department of Nuclear Medicine, University Hospital Bonn, Venusberg Campus 1, 53127 Bonn, Germany

**Keywords:** Education, Undergraduate Education, Medical students, Career choice, Obstetrics, Gynecology

## Abstract

**Introduction:**

Choosing a medical specialty is a pivotal moment in a physician’s career, shaped by personal interests, clinical experiences, and professional interactions. Obstetrics and gynecology (OB-GYN) offers a unique blend of surgical and medical care focused on women’s health. Given the growing demand for OB-GYN specialists, understanding the factors that influence students’ decisions is essential for workforce planning. This study compares the factors influencing first-year and final-year medical students at the University of Bonn in their decision to pursue OB-GYN.

**Methods:**

A total of 325 medical students participated in this longitudinal study, with 112 first-year and 213 final-year students completing digital surveys. The surveys assessed interest in OB-GYN, the importance of work-life balance, night shifts, future income, and the option for outpatient care. Statistical analyses, including Chi-square and McNemar’s tests, were used to identify significant changes in perceptions between the two groups.

**Results:**

Interest in pursuing OB-GYN declined from 60% among first-year students to 32% among final-year students (*p* < 0.001). The number of students concerned about night shifts increased from 48% in the first year to 76% in the final year (*p* < 0.001). Work-life balance was a critical factor for 97% of final-year students compared to 80% of first-year students (*p* < 0.01). The potential for outpatient care grew in importance, with 90% of final-year students prioritizing it in their specialty decision (*p* < 0.001).

**Discussion:**

The findings show that clinical exposure, lifestyle considerations, and mentorship significantly impact students’ interest in OB-GYN. Notably, interest in the specialty declined sharply from 60 to 32%, underscoring the importance of addressing key deterrents such as concerns about work-life balance. These challenges suggest that educational reforms should focus on creating flexible career paths and enhancing mentorship opportunities to attract and retain OB-GYN specialists. By tackling students’ concerns about lifestyle and offering adaptable career options, the specialty can sustain interest and ensure a sufficient future workforce to meet women’s healthcare needs.

## What does this study add to the clinical work

**Table:** 

This study provides key insights into the factors that influence medical students' decisions to pursue a specialization in obstetrics and gynecology (OB-GYN), which has significant implications for clinical practice and workforce planning. By identifying the role that lifestyle factors such as night shifts, work-life balance, and the appeal of outpatient care play in shaping career choices, this research can inform targeted interventions to address the challenges that deter students from choosing OB-GYN. Understanding these influences can support the development of flexible career options, part-time work opportunities, and mentorship programs designed to mitigate concerns about the demanding nature of OB-GYN practice. From a clinical perspective, ensuring a well-prepared and motivated OB-GYN workforce is critical for meeting the increasing demand for women's healthcare services. This study highlights the need for clinical institutions to promote work-life balance, provide structured mentorship, and create supportive environments that encourage medical students to enter and remain in the field of OB-GYN. By addressing these factors early in medical education, healthcare institutions can help attract a diverse and capable workforce, improving patient outcomes and enhancing the quality of care in obstetric and gynecologic services.	

## Introduction

The choice of medical specialization is a pivotal moment in the career of any physician, reflecting a combination of personal interests, career aspirations, exposure to clinical experiences, and mentorship. Among the numerous disciplines within medicine, gynecology and obstetrics (OB-GYN) hold a unique position, integrating both surgical and medical care focused on women’s reproductive health [1]. It is a field that combines high-stakes, acute interventions such as delivering babies and managing gynecologic emergencies with long-term, continuity-of-care for women throughout their lifespan. As such, OB-GYN attracts a distinct subset of medical students, whose motivations and decisions to pursue this specialty may evolve significantly over the course of their education [2].

The decision to specialize in OB-GYN is multifactorial and often develops throughout medical training, influenced by personal experiences, academic exposure, and professional interactions. Early-stage medical students, particularly those in their first semester, are generally in the process of exploring various fields and may not have firmly established preferences for any particular specialty. Their decisions are frequently shaped by preconceived notions about different specialties, societal influences, and limited clinical exposure [3]. Conversely, students nearing the end of their medical education, such as final-year students, have often had substantial clinical rotations and more direct exposure to OB-GYN [4]. These students have typically refined their career preferences based on hands-on experience and interactions with patients, mentors, and colleagues, potentially leading to a more informed and committed choice of specialization. Understanding how students’ preferences for a specialty like OB-GYN evolve from their first semester to their final year is of critical importance for medical education [5]. Early identification of factors that influence specialty choice could enhance the design of curricula and clinical experiences to better guide students toward the right career paths. Additionally, identifying trends in specialization preferences can help inform healthcare workforce planning, ensuring that sufficient numbers of students are choosing fields in which there may be future shortages, such as OB-GYN [6]. Given the increasing demand for women’s healthcare services globally, understanding the factors that influence the decision to specialize in OB-GYN is particularly relevant in addressing the potential challenges in the availability of specialists in this field.

Previous studies have investigated the factors that influence medical students’ choice of specialty, revealing a complex interplay of intrinsic and extrinsic motivations [7]. Intrinsic factors often include personal interest in the subject matter, perceived intellectual challenges, and a sense of personal calling to the field. In OB-GYN specifically, students may be drawn to the diversity of practice, the opportunity to care for women across their lifespan, and the combination of primary care and surgery within one specialty. Extrinsic factors, on the other hand, might include lifestyle considerations, financial incentives, perceived job satisfaction, and exposure to positive role models or mentors within the field [8].

For first-semester medical students, their understanding of OB-GYN as a potential career path is often shaped by preclinical coursework and any informal discussions they have had about the specialty [5]. At this stage, their interest may be primarily driven by a fascination with the reproductive system and the idea of participating in life-altering events such as childbirth. However, these students may also hold misconceptions about the field, including concerns about the long and often unpredictable hours, the emotional toll of managing high-risk pregnancies, or the perception that the specialty is predominantly for women, which may dissuade some male students. In contrast, students at the end of their medical studies have had substantial clinical exposure to OB-GYN through rotations, clerkships, and possibly elective experiences. This exposure allows for a more nuanced understanding of the realities of working in the field, including the breadth of conditions treated, the surgical and medical skills required, and the complexities of patient care in this domain. For these students, their decision to specialize may be more strongly influenced by concrete experiences, such as positive interactions with OB-GYN professionals, a sense of fulfillment from managing obstetric emergencies or gynecologic surgeries, or a particular affinity for patient populations served by this specialty. A key question in understanding specialty choice in OB-GYN is how preferences evolve over the course of medical school. Some students who express an early interest in OB-GYN may solidify that interest through clinical exposure, while others may shift their preferences as they gain more experience in other specialties or as they come to a more realistic understanding of the demands and rewards of the field. Furthermore, students who initially did not consider OB-GYN as a potential career may develop a newfound interest in the specialty as a result of positive clinical experiences, mentoring relationships, or a growing appreciation for the types of patient care and procedures that define the specialty.

The factors that lead to a change in specialty preference are likely multifactorial [9]. Clinical exposure is a dominant factor, as it allows students to experience first-hand the day-to-day realities of different medical disciplines. Additionally, personal life experiences, such as starting a family, or shifts in personal values during medical school, such as a growing desire for work-life balance, can also play a role. Peer and mentor influences are particularly important in OB-GYN, where strong role models who demonstrate a passion for the field can inspire students to pursue it as a career. Conversely, negative experiences, such as burnout or witnessing difficult patient outcomes, may lead students to reconsider their initial interest in OB-GYN.

The primary objective of this study is to compare the factors that influence the decision to specialize in OB-GYN between first-semester medical students and those at the end of their studies. By examining the differences in motivation, perceptions, and influences at these two stages of medical education, we aim to provide insights into how medical students’ career preferences evolve over time. Additionally, this study seeks to identify specific factors that may encourage or discourage students from pursuing a career in OB-GYN and to explore potential interventions that could be implemented early in medical education to support students in making informed, confident career decisions. Understanding these dynamics is essential for ensuring a well-prepared and motivated OB-GYN workforce capable of meeting the future needs of women’s healthcare.

## Methods

The University of Bonn Graduate Level Entry Medical School stands out within the German medical education landscape by admitting only graduate-entry students into its accelerated four-year medical program. This structure is distinctive compared to other German institutions, which often combine graduate-entry pathways with traditional undergraduate medical education. Graduate-entry students at Bonn are generally older, possess prior academic or professional experiences, and may exhibit different motivations, learning strategies, and career aspirations than their younger, undergraduate peers. This study sought to investigate the perceptions of obstetrics and gynecology (OB-GYN) among this cohort of graduate-entry students, aiming to better understand the factors shaping their career decisions, particularly in the context of their clinical exposure to OB-GYN.

### Educational context

At the University of Bonn, medical education culminates in an intensive clinical training period during the final year, including a mandatory one-week rotation in OB-GYN at the University Hospital Bonn. This hospital is a key regional provider of obstetric, midwifery, and neonatology services, offering a comprehensive environment for hands-on learning. In 2021 alone, the hospital facilitated 3030 live births, underscoring its role in regional healthcare and its importance in the training of future OB-GYN professionals. In Germany, specializing in OB-GYN requires robust training in both obstetrics and gynecology, reflecting the dual focus of the specialty. This rotation provides students with critical exposure to the clinical realities of these fields, potentially influencing their decisions to pursue a specialization in OB-GYN.

### Participants

**Fig. 1:**
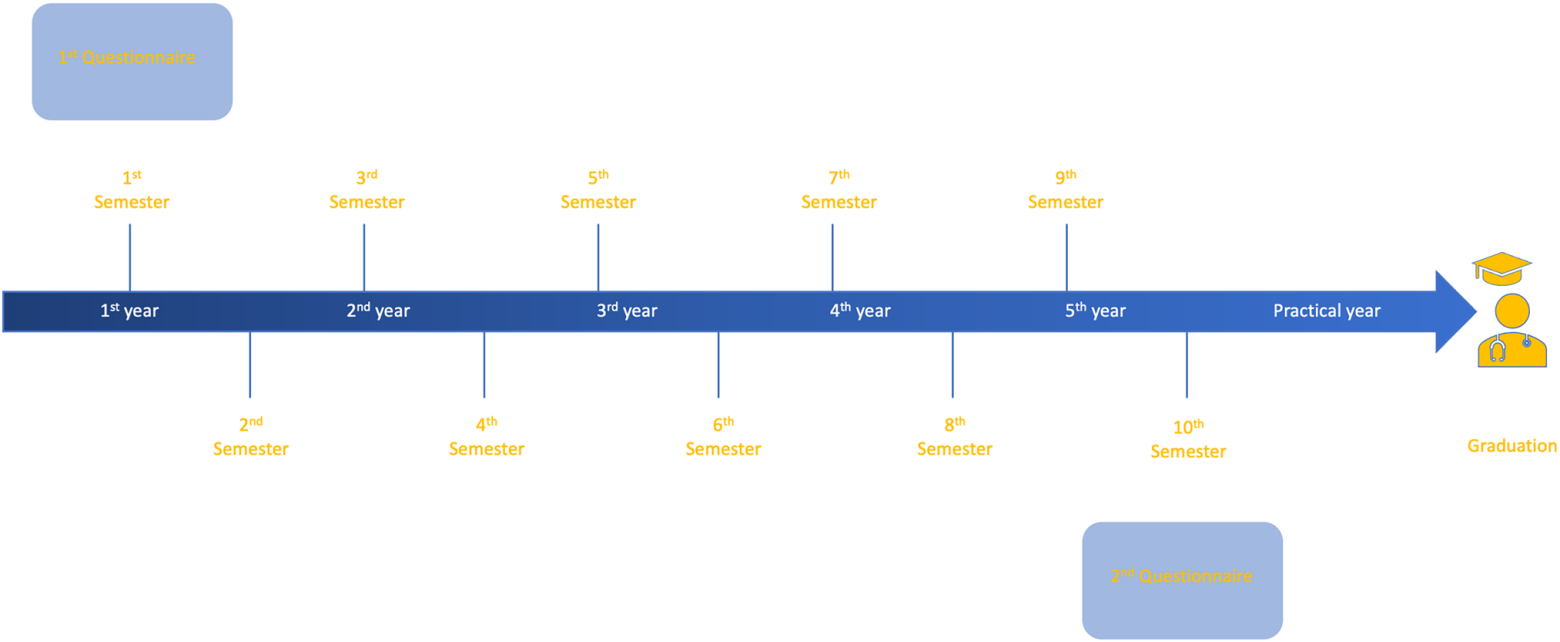
Symbolical and brief overview of how medical school is structured at the University of Bonn. Questionnaires were handed out during the first and last semester, with an average of 5 years between both point of times

The study targeted all first-year (*n* = 112) undergraduate students and all fifth-year (*n* = 213) undergraduate medical students at the University of Bonn. It was conducted between October 2023 and July 2024. Students were invited to participate in the study at two key time points: first, at the beginning of their medical studies (First year) and second, after completing their rotation and their Objective Structured Clinical Examination (OSCE) in OB-GYN (Fig. 1). Invitations were sent via personalized email, providing students with access to the study surveys through a QR code that linked them to the digital platform Evasys. This platform is regularly used for educational evaluations at the University of Bonn to assess and improve teaching quality. Participation in the study was voluntary and non-participation carried no academic penalties.

This longitudinal design allowed researchers to capture potential shifts in students’ perceptions of OB-GYN over the course of their clerkship and subsequent OSCE, offering insights into how practical experience might influence career aspirations.

### Measures

The study employed two digital surveys developed specifically for this research: a 9-item questionnaire for first-year medical students and a 17-item post-clerkship questionnaire for all fifth-year students. The surveys were designed with input from a multidisciplinary team of residents, medical students, and data analysts at the University of Bonn. The questions were informed by existing literature and previously validated instruments where applicable, although few pre-existing surveys addressed the specific aims of this study. Both questionnaires consisted primarily of dichotomous (yes/no) questions focused on factors influencing career decisions, such as interest in OB-GYN, work-life balance, expected future income, and family compatibility. Additional multiple-choice questions assessed preferred subspecialties within OB-GYN, as well as perceived positive and negative aspects of the specialty. One rating-scale question asked students to evaluate the educational value of their clerkship on a scale from 1 to 10.

To ensure consistency and facilitate comparison between pre- and post-rotation responses, the surveys shared a similar structure. These questions were aimed at capturing the students’ reflections on their clerkship experience, their perceptions of the specialty after clinical exposure, and any shifts in their career preferences. The study sought to provide a comprehensive view of how graduate-entry students at the University of Bonn perceive OB-GYN as a potential career, and how an immersive clinical rotation might influence their aspirations.

### Ethical approval

Ethical approval for the study was granted by the Faculty of Medicine Research Ethics Committee at the University of Bonn (EP 309/23). To maintain confidentiality and protect student anonymity, a gatekeeper was employed to handle the distribution, collection, and anonymization of identifying data before it was accessed by the research team. This process ensured compliance with ethical standards and minimized the risk of bias, as the researchers involved in supervising and grading the students did not have access to any identifying information.

### Statistical analysis

The data collected from both surveys were analyzed using a combination of descriptive and inferential statistical methods. Categorical variables were summarized using counts and percentages, while numeric data were tested for normality. For non-normally distributed data, the median and range were reported. McNemar’s test was used to assess differences in paired nominal data between the pre- and post-rotation responses, providing insights into changes in perceptions following the clinical experience.

To compare the responses of students at the beginning of their clerkship with those after their OSCE, Chi-square tests were conducted to identify statistically significant differences. Effect sizes for these comparisons were calculated using the phi coefficient (φ), allowing for the evaluation of the practical significance of the findings [10]. A significance level of 5% (*p* < 0.05) was used for all statistical tests, and no adjustments were made for multiple testing. All statistical analyses were performed using SPSS for Windows, Version 25.

By examining the shifts in attitudes and perceptions among graduate-entry medical students at the University of Bonn, this study aimed to provide a deeper understanding of the factors that influence their career choices, particularly with regard to specialization in obstetrics and gynecology.

## Results

The study included 325 students in total, with 112 in the first-year undergraduate student cohort and 213 in the last year undergraduate group. Their responses were analyzed for statistical significance to uncover trends and shifts in their attitudes toward OBGYN as a medical specialty and the most important factors influencing their decisions. The median age in the first-year cohort was 19 years (SD = ± 2.19 years); in the final year students were 25 years (SD = ± 3.83).

### The role of nightshifts in OBGYN

**Fig. 2:**
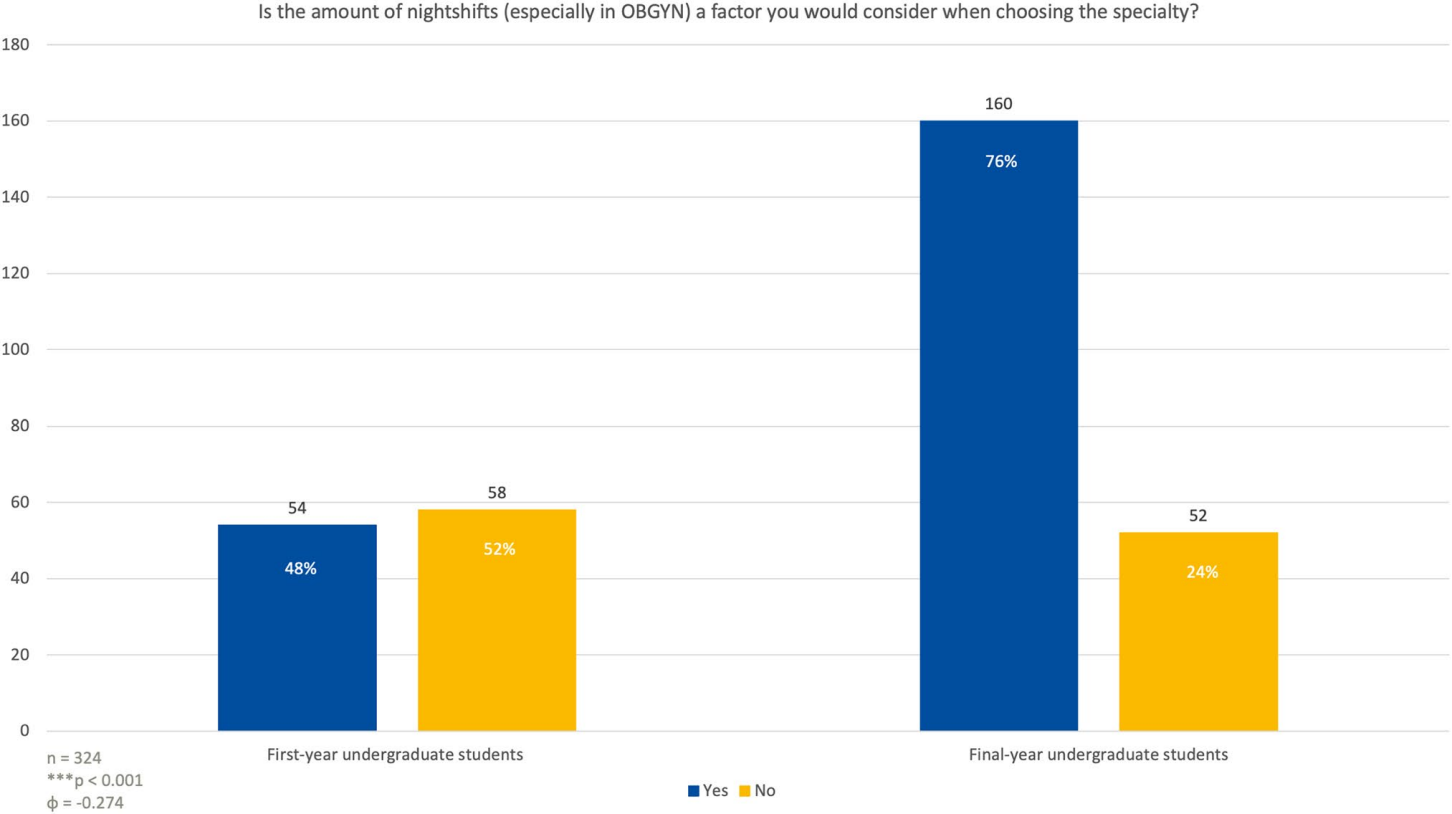
Students were asked if the amount of night shifts in OBGYN has an impact on their future career choice

When asked if the number of nightshifts, particularly in the field of OBGYN, would influence their decision when choosing a specialty, 54 first-year students, representing 48% of the group, indicated that it would be a factor (Fig. 2). In contrast, 58 first-year students, or 52%, said it would not play a role. Among last year students, a significantly higher proportion, 160 students (76%), reported that the number of nightshifts would indeed influence their decision, while 52 students (24%) stated it would not (*p* < 0.001; *φ* = 0.274).

### Working in outpatient care

**Fig. 3:**
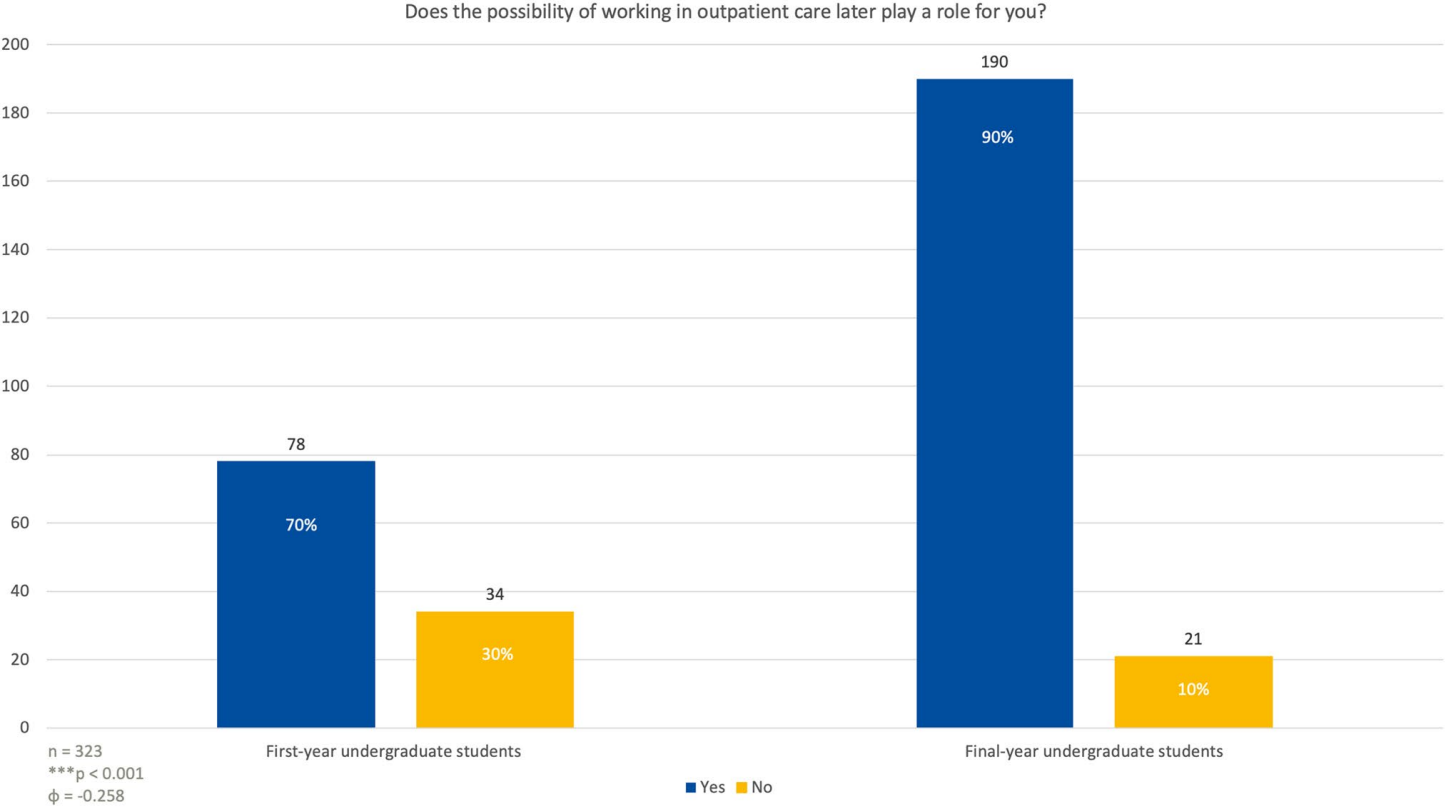
Students involved in the study were asked about the importance of having the possibility to work in outpatient care (e.g. working in private practice)

Regarding whether the option of working in outpatient care later in their careers would impact their choice of specialty, 78 first-year students, accounting for 70%, responded that this possibility would be important in their decision-making (Fig. 3). On the other hand, 34 first-year students (30%) said it would not play a role. A larger percentage of last year students, 190 individuals (90%), stated that the potential to work in outpatient care would influence their choice, while only 21 students (10%) said it would not affect their decision (*p* < 0.001; *φ* = 0.258).

### The impact of future income

**Fig. 4:**
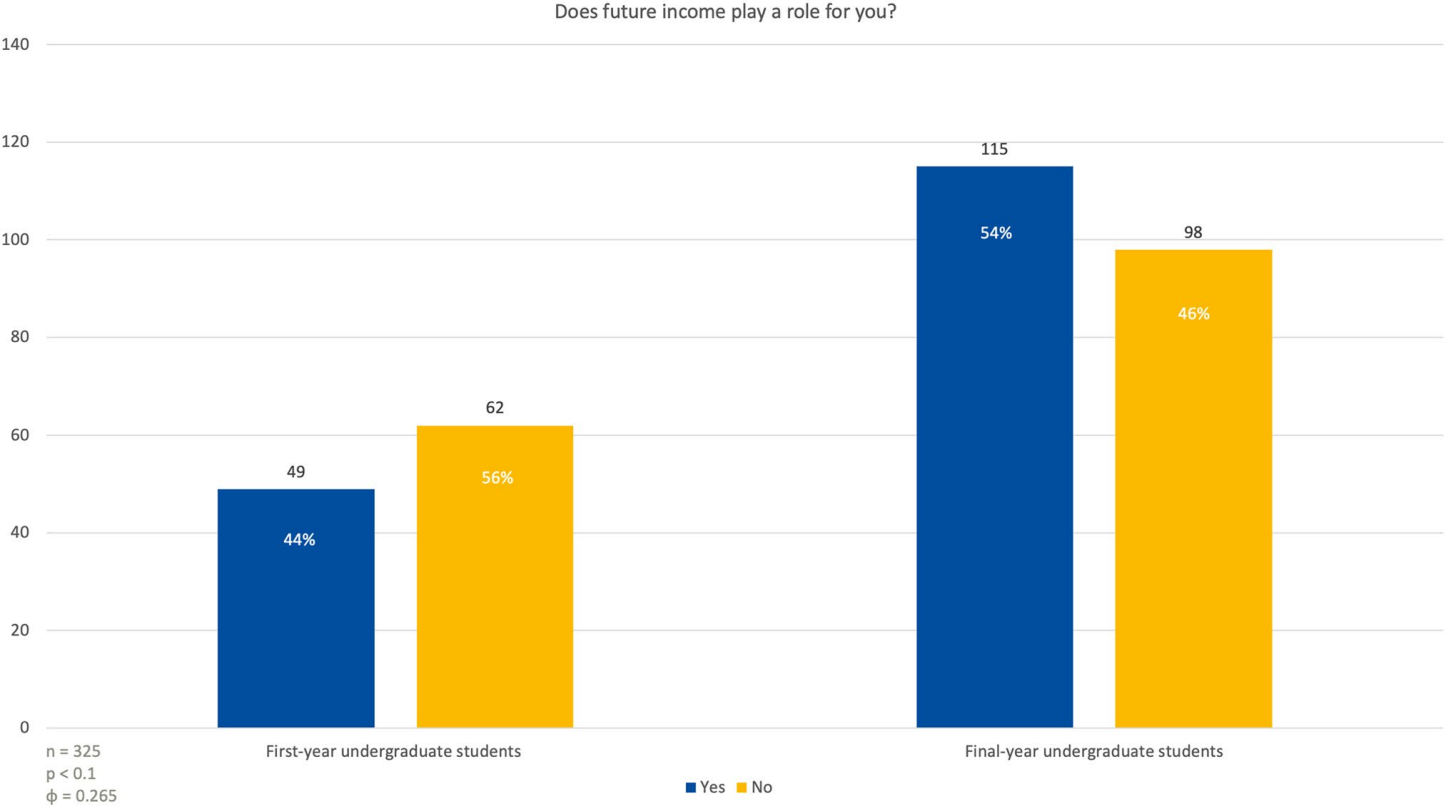
First and fifth-year students were asked to what extent salary influences their decision when selecting a medical specialty for their future career path

In response to the question of whether future income would be an important factor in selecting a medical specialty, 49 first-year students (44%) indicated that income would play a role in their decision, while 62 students (56%) said it would not (Fig. 4). Among last year students, 115 individuals (54%) considered future income to be an important factor, whereas 98 students (46%) did not see it as a significant consideration when choosing their specialty (*p* < 0.1; *φ* = 0.265).

### The importance of work-life balance

**Fig. 5:**
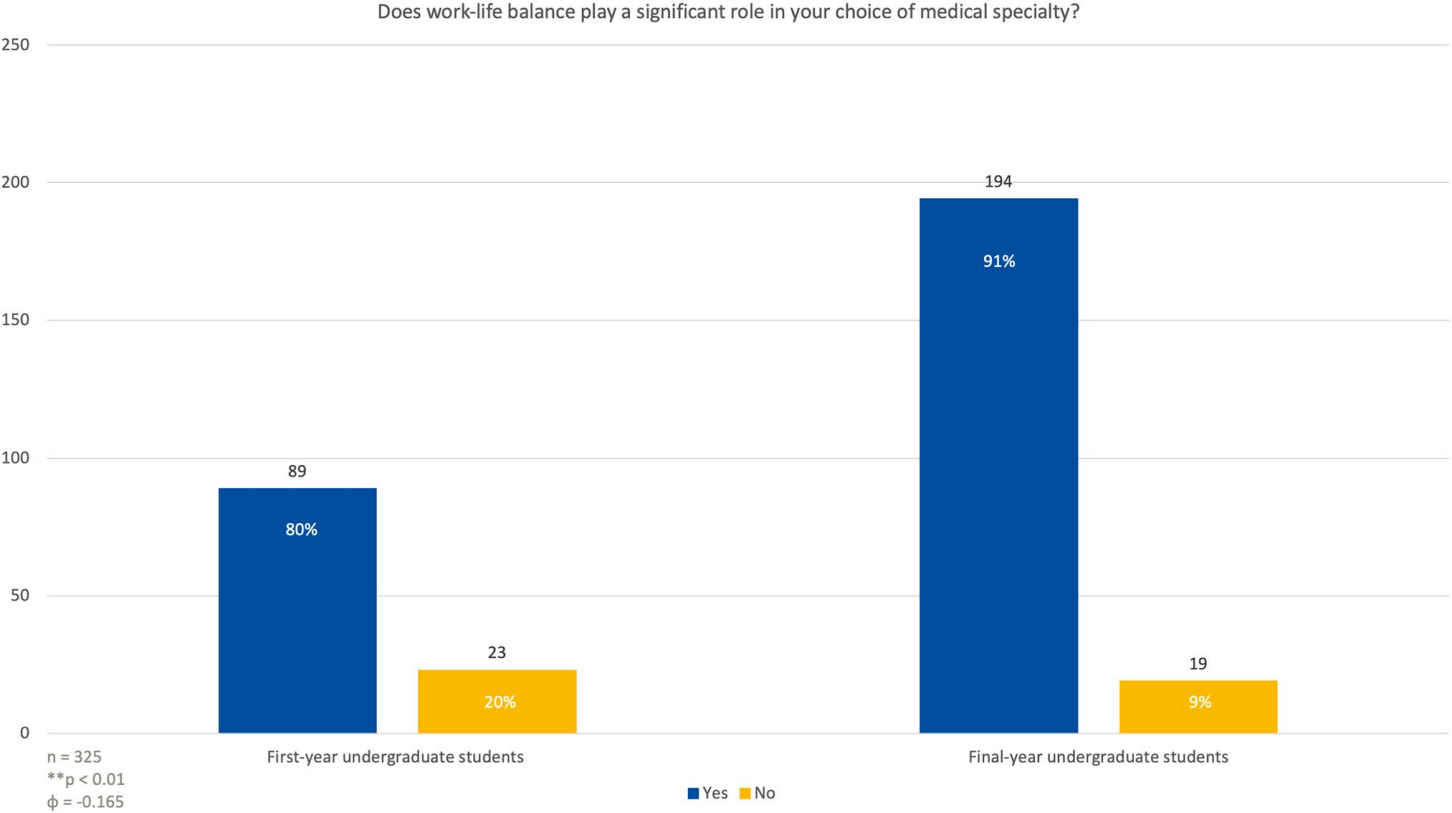
Medical students were asked how significant a factor work-life balance is when choosing a medical specialty in the future

When asked if achieving a balance between work and personal life was an important consideration in selecting a medical specialty, 89 first-year students (80%) responded affirmatively, stating that work-life balance was a significant factor in their decision. In contrast, 23 students (20%) said it was not a key consideration (Fig. 5). Among last year students, an overwhelming majority, 194 individuals (97%), indicated that work-life balance was crucial, while only 19 students (3%) felt it did not play a role in their decision-making process (*p* < 0.01; *φ* = 0.165).

### Aspiration to pursue a career in obstetrics and gynecology

**Fig. 6:**
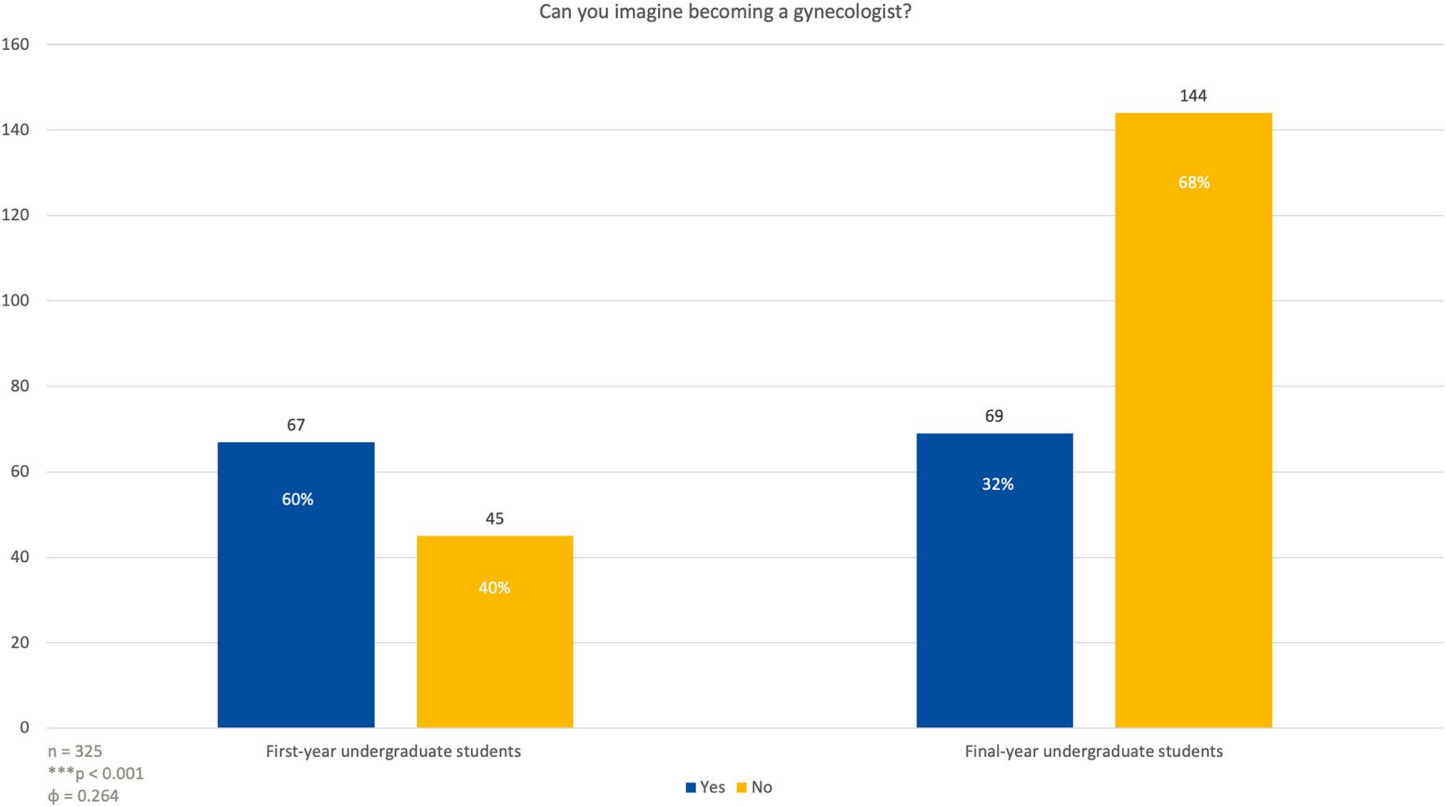
First-year and last-year students ‘ answers to the question if they would consider becoming an OBGYN after finishing medical school. Among first-year students, only 20% (*n* = 8) of males compared to 79% (*n* = 58) of females could envision themselves becoming gynecologists. By the final year, these figures shifted to 17% (*n* = 13) for males and 40% (*n* = 56) for females

When asked if they could envision themselves becoming gynecologists in the future, 67 first-year students (60%) said they could imagine themselves in this role, while 45 students (40%) said they could not (Fig. 6). In the last year group, 69 students (32%) responded that they could see themselves becoming gynecologists, whereas 144 students (68%) stated that they could not (*p* < 0.001; *φ* = 0.264).

**Fig. 7:**
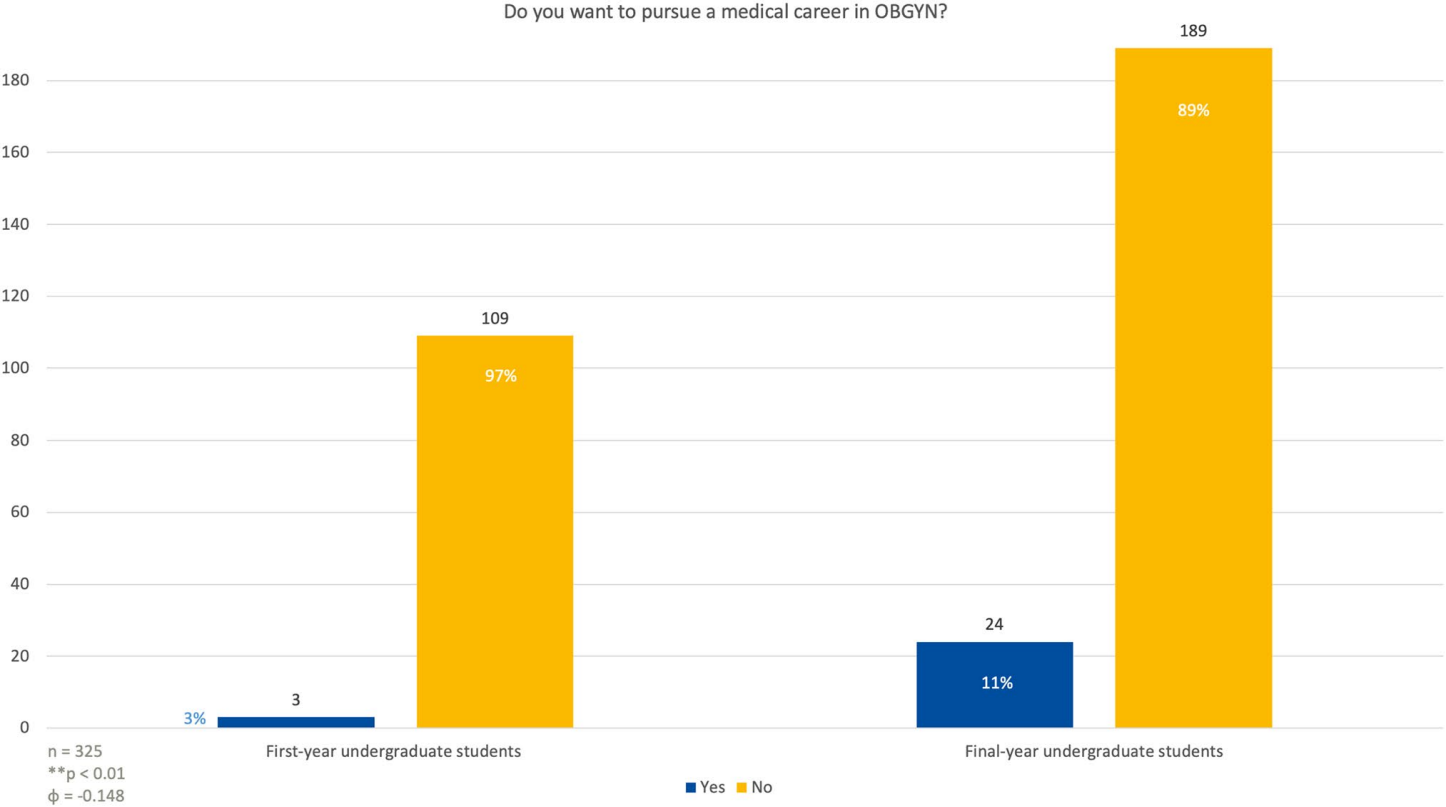
Students were asked if they are determined to pursue a career in OBGYN specifically. Among first-year students, none of the males were certain about pursuing a career in obstetrics and gynecology, while 4% (*n* = 3) of females reported they had already made up their minds. By the final year, 4% of males (*n* = 3) and 15% of females (*n* = 21) expressed a definite intention to pursue a career as gynecologists

In response to the question of whether they wished to pursue a career specifically in OBGYN, only 3 first-year students (3%) expressed a desire to do so, while the vast majority, 109 students (97%), said they were not interested in following this career path (Fig. 7). Among the last year students, 24 individuals (11%) expressed interest in pursuing a career in OBGYN, while 189 students (89%) stated that they did not wish to follow this specialty (*p* < 0.01; *φ* = 0.148).

## Discussion

The findings of this study reveal critical insights into the factors influencing medical students’ decisions to pursue a specialization in obstetrics and gynecology (OB-GYN) and how these factors evolve throughout their education. As the demand for OB-GYN specialists grows, understanding how students’ perceptions and preferences change, particularly as they gain more clinical experience, becomes essential for workforce planning, medical education reforms, and strategies to address potential shortages in women’s healthcare services. This discussion delves into the primary themes identified in this study: the impact of night shifts and lifestyle considerations, work-life balance [11], the appeal of outpatient care, the role of clinical exposure and mentorship, gender dynamics, and potential implications for medical education and workforce planning. Each theme is explored in relation to recent scientific literature to provide a more comprehensive understanding. The findings from this study show a significant shift in students’ perceptions of the importance of night shifts in choosing a specialty. In the early stages of their medical education, only 48% of first-year students considered night shifts as a determining factor, compared to 76% of fifth-year students. This change highlights the growing awareness of the practical realities of OB-GYN as students gain more clinical exposure, especially during rotations.

Recent studies confirm that lifestyle factors, including night shifts and long working hours, are increasingly important to medical students when deciding on a specialty. One systematic review by Lambert et al. (2018) found that lifestyle considerations were among the top three factors influencing specialty choice, particularly for specialties with high levels of intensity and frequent on-call shifts, such as OB-GYN and emergency medicine. This aligns with the observations in this study that final-year students, after experiencing clinical rotations in OB-GYN, are more likely to cite night shifts as a significant concern [12]. The demanding nature of OB-GYN, which often includes unpredictable working hours and frequent overnight shifts due to labor and delivery responsibilities, may lead students to reconsider their initial interest in the specialty.

Further, OB-GYN has been consistently ranked as one of the more demanding specialties in terms of lifestyle, which contributes to higher burnout rates compared to other fields. A study by Peckham et al. (2019) reports that 56% of OB-GYN residents experience burnout, largely attributed to long hours, night shifts, and the emotionally taxing nature of the work. This growing awareness of the toll of night shifts and demanding schedules may explain the declining interest in OB-GYN by the end of medical school observed in this study [1].

Work-life balance emerged as a critical factor influencing specialty choice for both first-year and fifth-year students in this study, with an overwhelming 97% of final-year students indicating its importance. This reflects a broader trend seen in medical education over recent years, where work-life balance is prioritized more highly than in previous generations.

Studies have consistently shown that newer generations of medical students, particularly millennials and Generation Z, value work-life balance more than previous cohorts, often prioritizing it over financial incentives or professional prestige. This generational shift has been highlighted in multiple studies, including one by Borges et al. (2020), which found that lifestyle compatibility was the most important factor for students choosing primary care, a field known for offering better work-life balance.

For OB-GYN, maintaining a healthy work-life balance can be particularly challenging due to the specialty’s nature [13], which involves managing emergencies, surgeries, and obstetric care, often at unpredictable times. Female medical students, in particular, tend to prioritize work-life balance when choosing a specialty. A survey of medical students by Gelfand et al. (2018) revealed that female students were more likely to consider how a specialty might impact their family life and personal commitments. Given that OB-GYN is a specialty that has historically attracted a higher proportion of female physicians [14], addressing work-life balance concerns within the field is critical for retaining interest among students.

Despite the challenges, efforts to improve work-life balance in OB-GYN have been implemented in some healthcare systems, such as part-time practice options, job-sharing, and flexible work schedules. However, the perception that OB-GYN is incompatible with work-life balance remains a barrier for many students, as seen in this study.

The preference for outpatient care as a factor in choosing OB-GYN grew significantly among fifth-year students [15], with 90% indicating that the opportunity to work in outpatient care would influence their decision, compared to 70% of first-year students. This shift underscores a broader trend in modern healthcare, where the emphasis is increasingly on outpatient and preventive care.

Outpatient care offers several advantages that appeal to students seeking a better work-life balance. For one, it typically involves more regular hours, fewer night shifts, and less acute care compared to inpatient settings, which aligns with the lifestyle preferences of many medical students. In OB-GYN, outpatient settings can include services like routine gynecological exams, prenatal care, family planning, and menopausal management, all of which provide opportunities for continuity of care without the unpredictable hours associated with obstetric emergencies. The increasing demand for outpatient care also reflects the broader healthcare landscape. A study by Rosenblatt et al. (2021) found that outpatient visits for OB-GYN services have grown steadily over the past decade, with more women seeking preventive care and reproductive health services outside of hospital settings. The growing emphasis on outpatient services may make OB-GYN more appealing to students who are looking for specialties that allow for both meaningful patient interactions and a manageable lifestyle.

Clinical exposure during rotations plays a pivotal role in shaping students’ career preferences [16]. This study shows that while first-year students might express interest in OB-GYN, their final career choice is often influenced by their hands-on experiences in the field. Only 11% of final-year students expressed a clear interest in pursuing OB-GYN, down from 60% of first-year students who initially envisioned a career in the field.

Positive clinical experiences, such as managing successful deliveries or developing strong relationships with mentors, can solidify a student’s interest in OB-GYN [17]. However, negative experiences, such as witnessing burnout, long hours, or difficult patient outcomes, can deter students from pursuing the specialty. Research by Raj et al. (2020) highlights that medical students who have strong mentors in their desired specialty are significantly more likely to choose that field for their careers. Mentorship, therefore, plays a crucial role, particularly in demanding fields like OB-GYN, where role models can demonstrate how to navigate the challenges of the specialty while maintaining a rewarding career.

This study suggests that mentorship in OB-GYN could be improved, especially to address concerns about lifestyle, emotional stress, and burnout. Structured mentorship programs that provide students with insights into how OB-GYN professionals balance the demands of their careers could help retain more students in the field. OB-GYN has traditionally attracted more female students due to its focus on women’s health. However, this study reveals that interest in the field decreases significantly among both male and female students by the final year of medical school. Gender dynamics play an important role in the decision to pursue OB-GYN [11], particularly given the emotional and physical demands of the specialty.

Female students, in particular, may be drawn to OB-GYN early in their training due to the opportunity to provide care for women throughout their reproductive lives. However, the demanding nature of the specialty, particularly with respect to work-life balance and the emotional toll of managing high-risk pregnancies, may lead many students to reconsider their initial interest. A study by Cochran et al. (2019) found that female students were more likely to cite concerns about family life and emotional exhaustion as reasons for choosing other specialties over OB-GYN.

Efforts to recruit male students into OB-GYN have also faced challenges. Some male students perceive OB-GYN as a female-dominated specialty [18], which may discourage them from pursuing it. Addressing gender-related barriers and ensuring that both male and female students feel supported in their decision to pursue OB-GYN is essential for maintaining a diverse and well-rounded workforce in the specialty.

A notable example of a successful mentorship program specifically designed for OB-GYN is the one implemented for US military medical students [19]. This program has proven to be highly effective in supporting students through the American residency matching process, with participants reporting that it significantly enhanced their preparedness and confidence. The mentorship initiative not only provided valuable guidance in refining application materials such as CVs and personal statements but also included mock interviews, which were instrumental in helping students navigate the complexities of the match process. The feedback from participants underscores the program’s success, with nearly all mentees expressing that they would participate again and felt more competitive as applicants following their involvement. Just as the military mentorship program helped students feel more prepared for their residency applications, it also likely addressed many of the challenges that students face when considering OB-GYN as a career. By offering both professional guidance and emotional support, the program fosters a deeper connection to the specialty, which could contribute to better retention rates and increased interest in OB-GYN, especially in light of the declining interest seen among students by their final year of medical school. Thus, this successful mentorship program serves as a valuable model for other institutions aiming to enhance the appeal and accessibility of OB-GYN for both male and female medical students. Another study from the US demonstrates that mentorship programs in OB-GYN are effective in enhancing medical students’ learning experiences and career development. By pairing senior resident physicians with medical students during their OB-GYN clerkships, the program provided personalized guidance and instruction in both clinical skills and career advice. Students reported high satisfaction, with 84% indicating they would participate again and 83% expressing that mentorship would be beneficial in other clerkships [20]. Additionally, residents did not feel burdened by the mentoring responsibilities and were also eager to continue. This feedback highlights that structured mentorship fosters a supportive learning environment, addresses students’ educational and emotional needs, and strengthens their connection to the OB-GYN specialty.

Since clerkships are also a part of medical education in Germany, and students often have concerns about various aspects of the specialty, such as work-life balance, mentorship programs like the ones mentioned above could be highly beneficial for German medical students.

### Implications for medical education and workforce planning

The results of this study have important implications for medical education and workforce planning, particularly in addressing the growing demand for OB-GYN specialists. First, early career guidance and exposure to the full range of opportunities within OB-GYN, including outpatient care and subspecialties, could help address misconceptions about the specialty. Educational programs should also provide students with realistic expectations about the lifestyle demands of OB-GYN, while offering strategies for managing these challenges, such as flexible working arrangements.

Mentorship programs, particularly those that emphasize work-life balance and provide positive role models in OB-GYN, are also crucial [21]. Such programs could help maintain interest in the field and counteract the negative perceptions that may arise during clinical rotations. By highlighting how OB-GYN professionals can achieve rewarding careers while maintaining a balanced lifestyle, medical schools can help retain more students in the specialty.

To create a more inclusive environment for male medical students in OB-GYN, medical schools should focus on reshaping perceptions of the specialty by emphasizing its diversity and offering equal opportunities for practical experience. Ensuring male students have access to all aspects of OB-GYN, including exams and surgeries, while providing mentorship from male role models and fostering gender-neutral clinical training, could help address gender biases. Additionally, promoting the field’s flexible career paths, along with actively combating discrimination, would make the specialty more appealing and accessible for male students. Another effective strategy to attract male medical students to OB-GYN could be the implementation of a mentorship program featuring male residents. In this program, mentors would provide insight into the complexities of surgeries in OB-GYN, while also addressing and challenging any stereotypes students may hold about the specialty. Such a program could inspire more male students to choose the specialty as their final-year elective, fostering a deeper connection to the field and increasing the likelihood of them pursuing it further in their medical careers [22].

Regular feedback and research should also be encouraged to ensure continuous improvement in inclusivity.

In terms of workforce planning, the declining interest in OB-GYN among final-year students is concerning, particularly given the increasing demand for women’s healthcare services. Addressing the factors that deter students from pursuing OB-GYN, such as concerns about night shifts and burnout, is essential for ensuring an adequate supply of OB-GYN specialists in the future.

## Limitations

This study has several limitations that warrant consideration. First, it was conducted at a single institution, the University of Bonn, which may restrict the applicability of the findings to other German universities or abroad. The distinctive focus of the University of Bonn, particularly its orientation toward graduate-entry medical students, may have influenced the perspectives and preferences of the participants, rendering these findings less reflective of the experiences of undergraduate medical students or those in other educational frameworks. A further limitation lies in the use of self-reported questionnaire data, which is prone to various biases, including social desirability bias. Participants might have responded in ways they perceived as socially acceptable or aligned with expected norms rather than honestly reflecting their true opinions. Additionally, the use of paper-based surveys, while practical, may have contributed to issues such as incomplete answers or non-response bias, potentially skewing the results if certain groups were more or less likely to engage fully. Finally, an additional challenge in the evaluation stems from the lack of a longitudinal design, which prevents tracking individual changes in decision-making over time. This limitation was a deliberate trade-off to simplify the questionnaire and maximize student participation, thereby offering a broad snapshot of perspectives. Moving forward, future studies will address this limitation by implementing a longitudinal approach, enabling the analysis of how individual attitudes toward the specialty evolve and influence career decisions over time.

## Conclusion

This study sheds light on the evolving factors that influence medical students’ decisions to pursue a career in OB-GYN. While many students express interest in the field early in their training, concerns about lifestyle, night shifts, and work-life balance often lead to a decline in interest by the final year of medical school. Addressing these concerns through mentorship, curriculum design, and career flexibility is essential for attracting and retaining students in OB-GYN, particularly given the growing demand for specialists in women’s healthcare.

## Data Availability

No datasets were generated or analysed during the current study.
